# Studies on the Catalytic Properties of Crude Freeze-Dried Preparations of *Yarrowia lipolytica* Extracellular Lipases for Geranyl Ester Derivative Synthesis

**DOI:** 10.3390/biom11060839

**Published:** 2021-06-04

**Authors:** Karina Jasińska, Bartłomiej Zieniuk, Dorota Nowak, Agata Fabiszewska

**Affiliations:** 1 Department of Chemistry, Institute of Food Sciences, Warsaw University of Life Sciences-SGGW, 159c Nowoursynowska St., 02-776 Warsaw, Poland; karina.jasinska8@gmail.com (K.J.); agata_fabiszewska@sggw.edu.pl (A.F.); 2 Department of Food Engineering and Process Management, Institute of Food Sciences, Warsaw University of Life Sciences-SGGW, 159c Nowoursynowska St., 02-776 Warsaw, Poland; dorota_nowak@sggw.edu.pl

**Keywords:** antibacterial, antioxidant, biocatalyst, cryoprotectant, esterification, freeze-drying, lipase, *Yarrowia lipolytica*

## Abstract

The study aimed to evaluate the impact of selected factors of the freeze-drying process on the hydrolytic and synthetic activity of the extracellular lipases of *Y. lipolytica* KKP 379 and to attempt the use of the crude enzyme preparation as a biocatalyst in the synthesis of geranyl 4-hydroxyphenylpropanoate. Antioxidant and antibacterial properties of the geranyl ester derivative were also investigated in order to evaluate their usefulness as a novel food additive. The studies confirmed that freeze-drying was an effective method of dehydrating yeast supernatant and allowed for obtaining lyophilizates with low water activity from 0.055 to 0.160. The type and concentration of the additive (2–6% whey protein hydrolyzate, 0.5% and 1% ammonium sulphate) had a significant effect on the hydrolytic activity of enzyme preparations, while the selected variants of drying temperature during the freeze-drying process were not significant (10 °C and 50 °C). Low yield of geranyl 4-hydroxyphenylopropionate was shown when the lyophilized supernatant was used (5.3%), but the yield of ester synthesis increased when the freeze-dried *Y. lipolytica* yeast biomass was applied (47.9%). The study confirmed the antioxidant properties of the synthesized ester by the DPPH**•** and CUPRAC methods, as well as higher antibacterial activity against tested bacteria than its precursor with 0.125 mM MIC (minimal inhibitory concentration) against *L. monocytogenes*.

## 1. Introduction

Lipases (EC 3.1.1.3) belong to the hydrolase family and their physiological role is to hydrolyze triacylglycerols to diacylglycerols, monoacylglycerols, fatty acids and glycerol [1]. Lipase was first discovered by Claude Bernard in 1856 in pancreatic juice as an enzyme that hydrolyzed insoluble oil droplets and transformed them into soluble products [2]. Since then, lipases have traditionally been obtained from the pancreas, but the interest in lipases of other origins, mainly from bacterial or fungal culture, has increased [3].

Enzymes derived from microbial cells are often more industrially useful than those derived from plants or animals due to the high yield production, the possibility of easy genetic manipulation of host cell, independence of the synthesis from climate and seasons, as well as the rapid growth of microorganisms in inexpensive waste media. Microbial lipases are also more stable than the corresponding plant and animal enzymes and their production is more convenient and safer [1].

In the current study, the possibility of obtaining lipases produced by the yeast *Yarrowia lipolytica* was highlighted. Due to the growing interest in biotransformation reactions in the chemical industry, these enzymes are desirable biocatalysts. They have high chemo- and stereospecificity, that favor their use in pharmacy, food technology, agrochemistry and cosmetology [4].

*Y. lipolytica* is a microorganism with unique physiological properties. The yeast is characterized by high secretory activity, synthesizes citric acid, γ-lactones and enzymes, and are able to use many substrates, e.g., carbohydrates, alcohols and lipids as a source of carbon in the cultures [5].

There are two main fractions of lipases synthesized by *Y. lipolytica*: intracellular (lipases associated with the cell membrane and cell wall structures) and extracellular [6]. The research on the *Y. lipolytica* genome revealed 25 putative lipase genes. The Lip2p protein is the major extracellular lipase that has been isolated and characterized. In addition to the Lip2p enzyme, other lipases have also been identified: Lip7p, Lip8p, Lip9p, Lip11p, Lip12p, Lip14p and Lip18p [7]. In 2000, the *LIP2* gene was isolated from *Y. lipolytica* and it was shown that this gene encodes a precursor protein of 334 amino acids, and the Lip2p lipase is a 301 amino acid glycosylated processed protein of 38 kDa molecular mass [8].

Due to the fact that *Y. lipolytica* yeast produces lipases intracellularly and extracellularly, both post-culture fluid (supernatant) and yeast biomass can be used as a biocatalyst [9]. In industrial applications, free lipases are not preferred, primary due to their low stability or the difficulty of the enzyme reusing. These disadvantages can be overcome, e.g., by immobilizing the enzymes on appropriate carriers, as well as, their spray-drying or freeze-drying, which are based on removing water and reducing the water activity, can be used for their preservation [6,10,11]. Moreover, in industrial practice, apart from pure enzymes, preparations of crude supernatants are also used. The use of biocatalysts in this form allows to simplify and minimize the costs of the production process, and there is no need to isolate and purify proteins.

Our previous works which focused on lipase-catalyzed esterification have acknowledged that *Yarrowia*-based biocatalysts are useful in synthesis reactions of phenolic compounds derivatives. For the past few years, the supernatant and biomass-derived enzyme preparations were both useful in order to synthesize novel compounds aimed for food technology [9,12,13]. It has not yet been established whether terpenoid derivatives can be also synthesized by means of those biocatalysts. This paper examines the freeze-drying process of the raw post-culture fluid as a method to extend the durability of lipolytic enzymes synthesized extracellularly by the microbial cells. Moreover, there has been outlined the usefulness of lyophilization to reduce the water content in lipase preparations in order to use them in esterification reactions. The aim of the study was to assess the influence of selected parameters of the freeze-drying process on the hydrolytic and synthetic activity of lipases contained in the supernatant of *Y. lipolytica* KKP 379 yeast culture and an attempt to use the crude enzyme preparation as a biocatalyst in the synthesis of geranyl 4-hydroxyphenylpropanoate. The yield of ester synthesis catalyzed by the crude freeze-dried supernatant was compared with the yield of the reaction catalyzed by the freeze-dried *Y. lipolytica* yeast biomass and the commercial immobilized preparation of lipase B from *C. antarctica.* The evaluation of its antioxidant and antibacterial activities was carried out in order to propose the ester as a novel food additive. 

## 2. Materials and Methods

### 2.1. Materials

Chemicals were purchased from Avantor Performance Materials Poland S.A. (Gliwice, Poland) and Sigma-Aldrich (Poznań, Poland). Culture media components were purchased from BTL Sp. z o. o. (Łódź, Poland), and olive oil was acquired in the local supermarket in Warsaw (Poland). *p*-Nitrophenyl laurate used in the lipase activity measurements was synthesized in the Department of Chemistry, Institute of Food Sciences, Warsaw University of Life Sciences-SGGW [14].

A commercial preparation of immobilized lipase B from *Candida antarctica* (Sigma-Aldrich) was also used in the current study.

### 2.2. Microorganisms

The yeast strain *Y. lipolytica* KKP 379 was used for the experiments and was purchased from the Collection of Industrial Microorganisms of Institute of Agricultural and Food Biotechnology (Warsaw, Poland). *Bacillus subtilis* PCM 486, *E. coli* PCM 2057, *Klebsiella pneumoniae* PCM 1 and *Listeria monocytogenes* PCM 2191 were obtained from the Polish Collection of Microorganisms of the Institute of Immunology and Experimental Therapy, Polish Academy of Sciences (Wrocław, Poland).

### 2.3. Yeast Cultures

YPO medium (1% yeast extract, 2% peptone and 2% olive oil) with 0.1% Tween 80 as emulsifier was used for yeast cultivation. The 200 mL of the prepared medium, pH 5.0, was placed in flat-bottom flasks, which were sterilized, and then were inoculated by adding 0.1% (*v*/*v*) of a 24-h *Y. lipolytica* KKP 379 inoculum in YPD medium (1% yeast extract, 2% peptone and 2% glucose) and cultured on a rotary shaker (140 rpm) for 48 h.

### 2.4. Freeze-Drying of Supernatant and Biomass of Y. lipolytica KKP 379

The yeast biomass was separated from the culture fluid using a high-speed centrifuge (8000× *g*, 20 °C, 8 min; Sigma, Osterode am Harz, Germany). The obtained supernatant was portioned and selected cryoprotectants were added and finally poured into Petri dishes of a 90 mm diameter. The following amounts of selected molecules were added: 0.5% and 1.0% ammonium sulphate and 2.0%, 4.0% and 6.0% milk protein hydrolyzate (Resource Instant Protein, Nestlé, Warsaw, Poland; protein content 90 g/100 g of the product). Control samples without additives and yeast biomass were also lyophilized. The plates were frozen at −40 °C in an Irinox freezer (Corbanese, Italy) and then subjected to the freeze-drying process in the Christ Gamma 1-16 apparatus (Osterode am Harz, Germany). The material was placed on shelves with a temperature of 0 °C, which provided the heat necessary for sublimation in a contact manner [15]. Secondary drying was carried out at the temperature of 10 °C or 50 °C.

### 2.5. Determination of Water Activity in Lyophilisates

The water activity (a_w_) of the obtained freeze-dried enzyme preparations was measured at a temperature of 20 °C using a laboratory analyzer for water activity measurements—Hygrolab (Rotronic AG, Bassersdorf, Germany).

The influence of water activity of lyophilized enzyme preparations on their hydrolytic activity was also investigated. In order to obtain materials with various water activity, they were kept in desiccators with selected relative humidity (RH), obtained over saturated organic salt solutions. Desiccators with relative humidity of 0 (CaCl_2_), 0.329 (MgCl_2_), 0.529 (Mg(NO_3_)_2_) and 0.750 (NaCl) were used. The material was kept in desiccators by 1 week and then their hydrolytic activity was tested.

The hydrolytic activity of selected lyophilized enzyme preparations after storage at 0 RH for 4 months was also tested.

### 2.6. Determination of the Hydrolytic Activity of Freeze-Dried Supernatants

A modified spectrophotometric method of measuring the progress of the *p*-nitrophenyl laurate hydrolysis according to Kapturowska et al. [16] was used to evaluate the hydrolytic activity. Briefly, the hydrolysis was carried out in Erlenmeyer flask and 0.3 mmol of *p*-nitrophenyl laurate dissolved in 2 mL of heptane and 0.25 g of the freeze-dried preparation and 15 mL of distilled water were stirred at 37 °C. After 15 min of stirring absorbance was measured at 410 nm in UV/Vis spectrophotometer. The unit of lipase enzymatic activity was 1 U, i.e., the amount of enzyme that liberated 1 µmol of *p*-nitrophenol per minute under the assay conditions.

### 2.7. Geranyl 4-Hydroxyphenylpropanoate Enzymatic Synthesis

**Figure 1:**
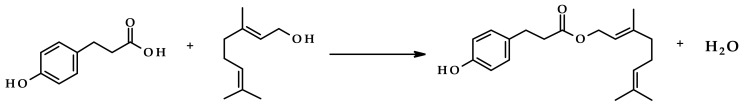
Scheme of geranyl 4-hydroxyphenylpropanoate synthesis.

In the current work, the ester synthesis from 4-hydroxyphenylpropanoic acid and geraniol were carried out according to Figure 1. The same substrates in a ratio of 1:1.5 (acid:alcohol) and the same solvent mixture (*tert*-butyl-methyl ether : isooctane, 1:1) were used for all syntheses reactions, but different biocatalysts were used: commercial preparation of lipase B from *C. antarctica*, the freeze-dried biomass of *Y. lipolytica*, and the crude freeze-dried supernatant (with 1% (NH_4_)_2_SO_4_).

Substrates (0.0025 moles of acid), biocatalyst (*C. antarctica* lipase B—5% *w*/*w* of substrates mass or freeze-dried *Y. lipolytica* preparations—2 g) and 20 mL of the solvent mixture were placed in flasks. Reactions were carried out in a rotary shaker (250 rpm) at 37 °C for 96 h. After the reaction, the resulting mixture was subjected to purification. Solvents were evaporated using Büchi Rotavapor R-200 (Büchi AG, Flawil, Switzerland), and then the residual was dissolved in approx. 10 mL of chloroform and placed on magnetic stirrers for 30 min with the addition of 8 mL of 5% sodium bicarbonate. Then, the organic phase was separated, and magnesium sulphate was added. Finally, the drying agent was filtered off and the solvent was evaporated. Ester was purified using column chromatography, and silica gel 60 (0.040–0.063 mm; 230–400 mesh) was used as a stationary phase and chloroform : methanol (9:1) were applied as a mobile phase. Fractions were collected in separate flasks and then analyzed by TLC. Those containing ester were then dried and the mixture of solvents was evaporated.

The purified ester was subjected to nuclear magnetic resonance (^1^H NMR) spectroscopic analysis to confirm the structure of the compound. ^1^H NMR spectra were recorded on a Bruker AVANCE 300 MHz spectrometer (Bruker, Billerica, MA, USA) using CDCl_3_ as a solvent and tetramethylsilane (TMS) as an internal standard.

^1^H NMR (300 MHz, CDCl_3_): δ 1.63 (3H, s), 1.71 (6H, s), 2.05–2.20 (4H, m), 2.61 (2H, t, *J* = 7.7 Hz), 2.90 (2H, t, *J* = 7.7 Hz), 4.61 (2H, d, *J* = 7.1 Hz), 5.06 (1H, s), 5.33 (1H, t, *J* = 7.7 Hz), 5.45 (1H, t, *J* = 7.7 Hz), 6.71–6.82 (2H, m), 7.03–7.13 (2H, m).

### 2.8. Evaluation of Antioxidant Activity of Geranyl 4-Hydroxyphenylpropanoate

The antioxidant activities of the synthesized ester, its precursor and geraniol were compared by means of the DPPH**•** radical method and the CUPRAC method [17]. The percentage reduction of the DPPH**•** radical after 60 min and the IC_50_ parameter, referring to the concentration required for 50% reduction of the DPPH**•** radical were determined. In the latter method Trolox equivalent antioxidant capacities (TEAC) were determined for the tested compounds.

### 2.9. Evaluation of Antiobacterial Activity of Geranyl 4-Hydroxyphenylpropanoate

The minimum inhibitory concentration (MIC) of ester and its precursor—4-hydroxyphenylpropanoic acid—was determined by the microdilution broth method according to ISO against 4 bacterial strains [18]. Furthermore, minimum bactericidal concentrations (MBC) were determined. Briefly, 3 µL of bacterial culture from each well without observed growth was transferred onto Mueller–Hinton Agar. Subsequently, agar plates were incubated at 37 °C for 24 h, and the growth of microorganisms was checked, which indicate the MBC values.

### 2.10. Calculation of Lipophilicity Properties of Tested Compounds

cLogP (partition coefficient) and cLogS (logarithm of S, where S is water solubility in mol/L, pH = 7.5, 25 °C) of the tested compounds were calculated with Osiris DataWarrior (DataWarrior V5.0.0, Idorsia Pharmaceuticals Ltd., Allschwil, Switzerland).

### 2.11. Statistical Analysis

Statistical analysis was performed using Statistica 13.3 software (TIBCO Software Inc., Palo Alto, CA, USA). In order to verify the statistical hypothesis of the normality of the distribution of experimental data, the Shapiro–Wilk test was used, and the Levene’s test was used to check the hypothesis of the homogeneity of variance. The results were analyzed using one-way analysis of variance (ANOVA), Tukey’s post hoc test and for the remaining ones, a non-parametric test—the Kruskal–Wallis test, was used. The significance level was α = 0.05.

## 3. Results and Discussion

### 3.1. The Impact of Water Activity in the Crude Freeze-Dried Enzyme Preparations on Their Hydrolytic Activity

Water is necessary to protect the catalytic conformation and functionality of the biocatalyst. It is an essential element to maintain the integrity of the three-dimensional structure of the enzyme molecule, and its sufficient amount is needed to initiate the chemical reaction. The control of water content and water activity is also important due to the fact that the direction of catalysis of lipases is based on the thermodynamic equilibrium between the hydrolysis and esterification reactions [19]. At high water content in the reaction medium, lipases will catalyze ester bond hydrolysis reactions, and low water content and water activity will predispose proteins to catalyze esterification and transesterification reactions in organic solvents [4,20]. The effectiveness of the freeze-drying process is demonstrated by the water activity of the tested enzyme preparations. During freeze-drying, yeast cells lose at least 80–85% of their water, going into the state of anabiosis, which increases their survival in difficult conditions [21]. Despite the loss of water, lyophilized yeast cells still contain a certain amount of water, bound in various ways with the cell structures, which allows them to ensure the catalytic activity of the enzymes.

In the current research, it has been shown that all the obtained crude freeze-dried supernatants were characterized by low water activity parameter in the range of 0.050–0.160 (Table 1), which indicated the possibility of their use as biocatalysts in chemical reactions.

**Figure 2:**
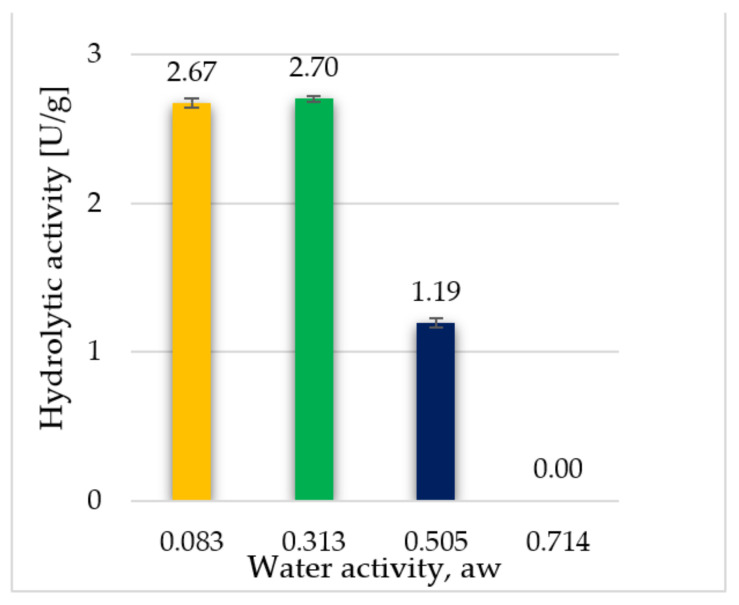
Influence of water activity (a_w_) on the hydrolytic activity of freeze-dried enzyme preparations with the addition of 1% ammonium sulphate. Experiments were performed in triplicate.

In the next stage, an experiment was conducted to assess how the water activity of freeze-dried enzymes influences their hydrolytic activity. Table 2 shows the potentially set value of water activity that prevailed in a given desiccator with the actual measured result of water activity for the freeze-dried supernatants just after removing the preparations. Although some preparations were stored in an environment with water activity equal to 0, in fact, the value of water activity in the lyophilisate itself was slightly higher, which ensured that the enzyme activity was maintained (2.67 U/g, and 2.70 U/g for the preparation stored in a desiccator with a water activity of 0.329). It was observed that there was no statistically significant difference between the hydrolytic activity of enzyme preparations with water activity 0.083 and 0.313 (Figure 2).

The increase in the water activity of enzyme to 0.505 resulted in a 2.5-fold decrease in the hydrolytic activity of the enzymes present in the preparation, while the dried supernatants at the water activity of 0.714 turned out to be inactive.

It is important to adjust the amount of water needed to maintain the catalytic activity of the enzyme. The optimal range can often be very narrow and depends on the biocatalyst itself. It is possible that high water activity could reduce the reaction rate by aggregating enzyme molecules and causing diffusion restrictions [20].

### 3.2. The Hydrolytic Activity of Crude Freeze-Dried Enzyme Preparations

**Figure 3:**
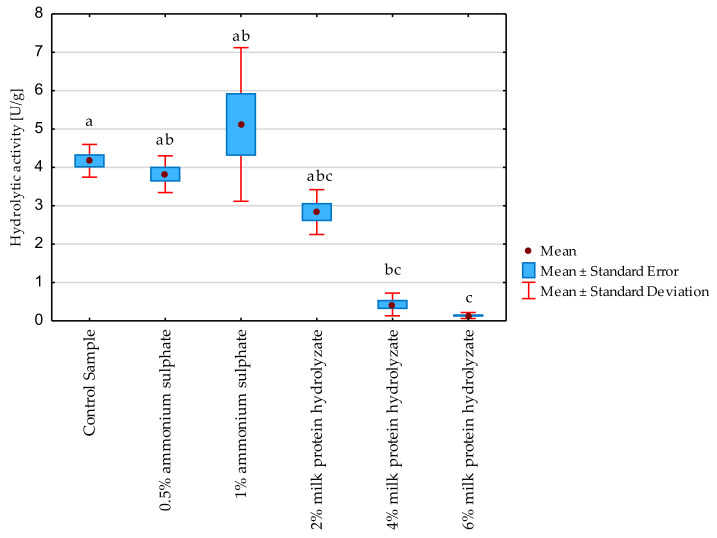
Effect of the used additive on the hydrolytic activity of freeze-dried preparations. Significant differences are marked with letters of the alphabet. Experiments were performed in triplicate.

Hydrolytic activity of crude freeze-dried preparations was determined. Firstly, the effect of the additives on hydrolytic activity was determined, and the results were confirmed by non-parametric tests (Figure 3). The following additives were used: 0.5% and 1% ammonium sulphate and 2, 4 and 6% milk protein hydrolysate (Table 1). Significant differences were observed between the control sample and the lyophilisate with the addition of 4 and 6% milk protein hydrolyzate. The abovementioned additives reduced the hydrolytic activity in relation to the preparation without any protective substance. In the case of the rest of the samples no statistically significant differences were observed.

**Figure 4:**
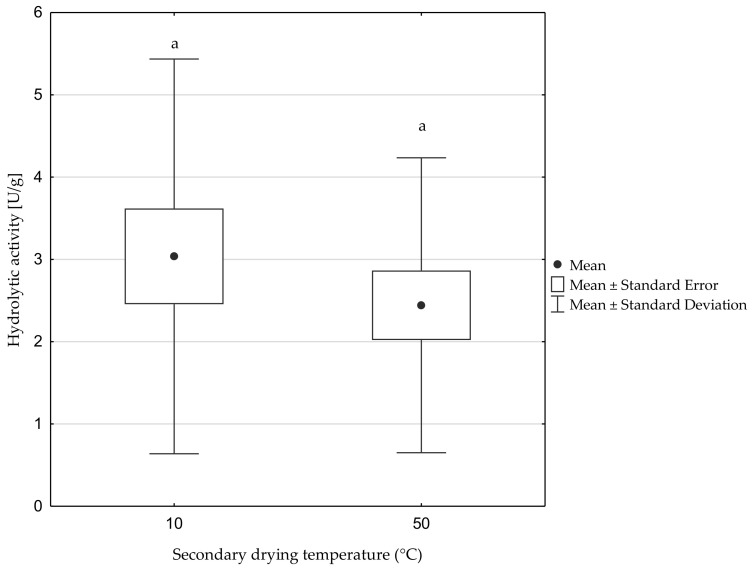
Effect of the secondary drying temperature on the hydrolytic activity of freeze-dried preparations. Significant differences are marked with letters of the alphabet. Experiments were performed in triplicate.

The second tested factor was the secondary drying temperature (10 and 50 °C). It was found that there were no significant differences between these values, and therefore the factor had an insignificant effect on the hydrolytic activity of the preparations (Figure 4).

**Figure 5:**
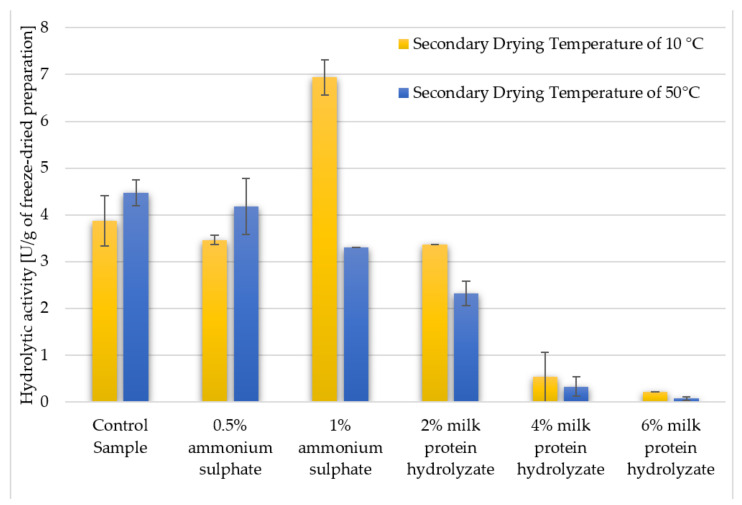
The effect of the applied cryoprotectant and the secondary drying temperature during the freeze-drying process on the hydrolytic activity of the lyophilized enzyme preparation (calculated per 1 g of the final preparation taking into account the presence of both the lyophilized supernatant and the addition of cryoprotectant). Experiments were performed in triplicate.

**Figure 6:**
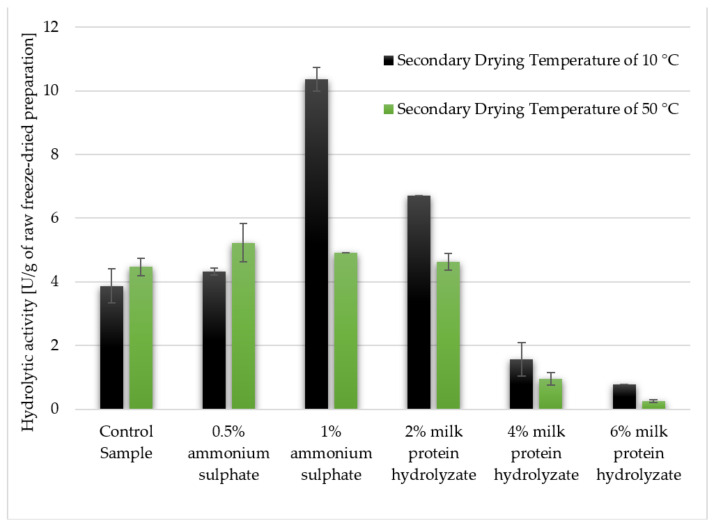
The effect of the applied cryoprotectant and the secondary drying temperature during the freeze-drying process on the hydrolytic activity of the lyophilized enzyme preparation (calculated per 1 g of the preparation, referring only to the lyophilized supernatant, excluding the presence of the cryoprotectant). Experiments were performed in triplicate.

By comparing the hydrolytic activity of the freeze-dried enzyme preparations (Figure 5 and Figure 6), it can be seen that the amount of additive is a significant factor. It can be concluded that the best variant turned out to be a 1% addition of ammonium sulphate, dried at 10 °C, and the worst was the preparation with 6% addition of milk protein hydrolyzate, dried at 50 °C. We managed to achieve two times higher lipolytic activity for the preparation with 1% ammonium sulphate and 10 °C secondary drying temperature than for the control samples and preparations made with the addition of 0.5% of the salt or prepared with higher secondary drying temperature. Considering the addition of ammonium sulphate, it can be seen that higher concentration positively influenced the hydrolytic activity, and the opposite tendency can be observed for the hydrolyzate of milk proteins, where, with increasing amounts, it led to a decrease in the hydrolytic activity of enzymes. Enzyme preparations with 4% addition of this cryoprotectant showed almost 10 times (0.53 U/g), and those with 6% milk protein hydrolyzate almost 20 times lower (0.21 U/g) value of activity compared to the control sample (3.86 U/g) in case of 10 °C, and these ratios increased to almost 15 and 65 when the secondary drying temperature was 50 °C and activities were 4.47, 0.32 and 0.07 U/g for the control sample, sample with 4% and 6% milk protein hydrolyzate addition, respectively (Figure 5). Similar remarks can be shown referring to the activities of tested samples when were calculated excluding the presence of the cryoprotectant (Figure 6). However, interestingly the sample with 2% addition of milk protein hydrolyzate dried at 10 °C exhibited higher activity (6.72 U/g) than control samples.

Although the milk proteins should be a good source of calcium ions for lyophilized enzymes [22] and provide a protective coat for enzyme molecules [23], with the increased amount of the additive in the preparation, they did not provide the positive effects on the maintenance of the activity after the process of freeze-drying. It is possible that the freezing temperature could led to stresses destabilizing the protein structure. Many works, where skim milk was used as a cryoprotectant, showed a positive effect of the additive on the lyophilized preparation and provided it with a uniform porous structure, enabling both easy drying of the sample and its subsequent rehydration [24]. Based on crystallographic studies, it has also been proven that lipases bind to calcium cations around the active site of the enzyme, which results in improved thermal stability as well as catalytic activity [25].

With regard to the additives used, it can be seen that 1% of ammonium sulphate had a positive effect on the hydrolytic activity of the freeze-dried enzyme preparations. The salt showed a stabilizing effect on proteins contained in samples. Our results are in line with other researchers [26,27]. Thus far, much work has been done on this issue and it has been divided into two areas. The first one involves the effect of relatively low concentrations of divalent ions, such as Ca^2+^, Mg^2+^, Zn^2+^, etc., on the thermostability of enzymes. This effect is very specific and therefore is not considered as a general method of enzyme stabilization. The second area deals with determining the effect of relatively high concentrations of various salts. They can bind to charged groups of proteins or dipoles or reduce the solubility of hydrophobic groups of proteins by increasing the ionic strength of the solution. Salting out the hydrophobic residues on the surface into the macromolecule of the enzyme conformationally compresses the enzyme. As a result, it becomes more resistant to thermal decomposition and may show higher thermal stability. With respect to this reversible process, the experimental data substantially correlates with the Hofmeister (lyotropic) series on the effect of salt on protein solubility. The stabilizing effect of cations decrease in the following order: (CH_3_)_4_N^+^ > NH_4_^+^ > K^+^, Na^+^ > Mg^2+^ > Ca^2+^ > Ba^2+^ and in anions: SO_4_^2-^ > Cl^−^ > Br^−^ > NO_3_^−^ > ClO_4_^−^ > SCN^−^ [26,27].

Analyzing the above series, it can be concluded that the beneficial effect of the presence of ammonium sulphate in the lyophilized preparation on the catalytic properties of lipases could be due to the above-described properties of this inorganic salt.

Another important factor that was considered in the context of its influence on the hydrolytic activity was the drying temperature in the freeze-drying process. The aim of desorption drying (also called secondary drying) is to remove the adsorbed water. This freeze-drying step must be adjusted individually to the thermosensitivity of each raw material. Typically, the drying temperature of biological materials is in the range from 10 to 35 °C for thermally sensitive materials and up to 50 °C for thermally resistant materials [28]. In the case of the obtained preparations, no significant differences were noted between the applied drying temperatures, which may indicate a relatively wide range of thermal sensitivity of the tested enzymes.

### 3.3. Hydrolytic Activity of Freeze-Dried Enzyme Preparations after Storage for 4 Months

Freeze-drying is considered a popular method of microbial protection that in most cases preserves the long-term viability of cells and their enzymes [29]. In the cultivation of microorganisms, apart from biomass, a post-culture fluid (supernatant) is obtained, whose storage may generate costs due to the size of the material. It is much easier to store the supernatant in the form of a lyophilisate, as long as carrying out the freeze-drying process does not affect the activity of the biocatalyst [6].

**Figure 7:**
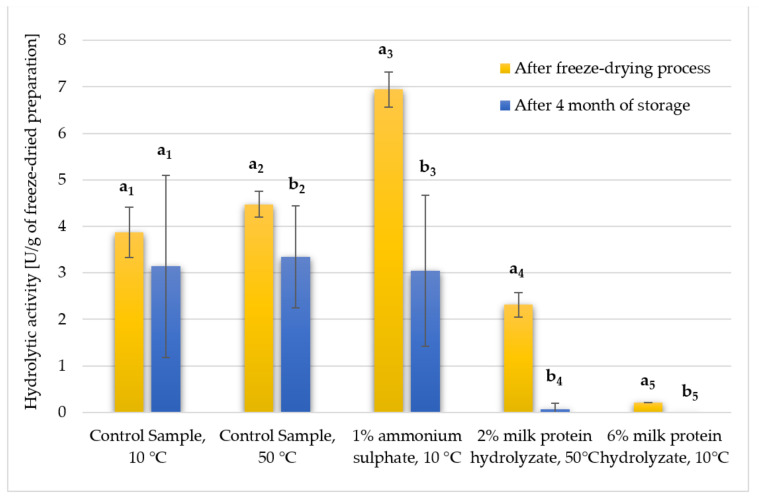
Hydrolytic activity of selected freeze-dried enzyme preparations after storage in a desiccator for 4 months (calculated per 1 g of the final preparation taking into account the presence of both the lyophilized supernatant and the addition of cryoprotectant). Significant differences are marked with letters and a number given in a subscript indicate the compared values. Experiments were performed in triplicate.

**Figure 8:**
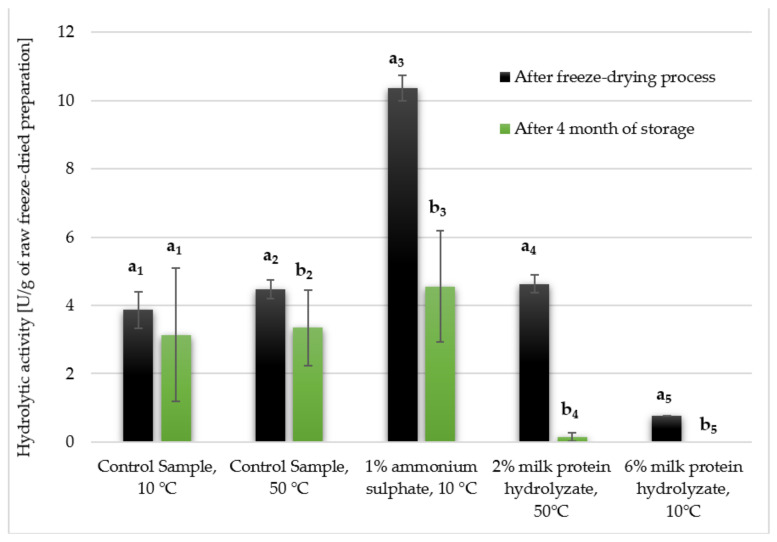
Hydrolytic activity of selected freeze-dried enzyme preparations after storage in a desiccator for 4 months (calculated per 1 g of the preparation, referring only to the lyophilized supernatant, excluding the presence of the cryoprotectant). Significant differences are marked with letters and a number given in a subscript indicate the compared values. Experiments were performed in triplicate.

The conducted studies assessed how the hydrolytic activity of selected enzyme preparations changed after storage in a desiccator after 4 months. In non-parametric tests, only one particular type of lyophilisate was compared to determine the effect of storage time (Figure 7 and Figure 8).

The control sample without additives dried at 10 °C turned out to be the most stable. The remaining lyophilisates showed statistically significant differences between the value of hydrolytic activity immediately after freeze-drying and that after storage for 4 months. The most significant decrease in the activity was noted for the preparation with the addition of 1% ammonium sulphate, where in the initial stage its activity was 6.94 U/g, and after storage for 4 months it decreased to 3.05 U/g. The freeze-dried samples with the addition of milk protein hydrolyzate did not survive, as for both concentrations the activity dropped to almost 0 U/g (Figure 7). The same observations were noted when the activity was calculated per 1 g of the preparation, referring only to the lyophilized supernatant, excluding the presence of the addition (Figure 8). Therefore, it can be concluded that the applied additives did not provide the expected durability of the enzymes contained in the preparation, which resulted in a significant reduction in their hydrolytic activity during storage.

### 3.4. Esterification of 4-Hydroxyphenylpropanoic Acid with Geraniol

Enzymatic productions of aromas, fragrances or compounds with antioxidant and antimicrobial properties have become the subject of scientific and industrial interest. The compounds that have also gained interest are geraniol ester derivatives due to their presence in the vast majority of essential oils such as rose and citronella exhibiting antimicrobial activity that can act also as a low-toxicity pesticide. Geranyl acetate has been marked by the American Food and Drug Administration with the GRAS status. The production of esters can be carried out by lipase-catalyzed synthesis and several esters, such as eugenyl acetate, benzyl acetate, cinnamyl propanoate, geranyl propanoate and citronellyl acetate were produced by this method [30,31,32].

In the current work, an attempt was made to synthesize the ester using geraniol and 4-hydroxyphenylpropanoic acid as substrates in order to obtain a compound with potential use as a food additive with antioxidant and/or antimicrobial activity. The synthesis was carried out with the use of three different types of biocatalysts. The highest synthesis efficiency was achieved when a commercial preparation of immobilized lipase B from *C. antarctica* was used (73.4%), then with lyophilized *Y. lipolytica* biomass (47.9%), and the lowest efficiency was observed for crude freeze-dried *Y. lipolytica* supernatant—5.3% (Table 3).

Although both biocatalysts derived from *Y. lipolytica* yeast cultures were freeze-dried, a higher yield was achieved for biomass, which may mean that the enzymes contained therein showed higher specificity to the substrates used. It is well-known that *Y. lipolytica* is capable of synthesizing intracellular enzymes such as lipases or esterases, which are found in the cytosol and are associated with the structures of the cell wall, as well as extracellular [33]. Similar observations were noted in the work of Zieniuk et al. [9], where the biomass of *Y. lipolytica*, unlike the freeze-dried supernatant, was an efficient catalyst for the synthesis of ethyl hydrocinnamate.

Differences between yields may be due to the presence of various types of enzymes in preparations, including esterases and proteases that can catalyze the same reactions as lipases. The conducted experiment showed that extracellular enzymes in the supernatant were not active enough to achieve a satisfactory level of substrate conversion. Despite the large differences in the efficiency of the reaction between the enzyme preparations of *Y. lipolytica* and CALB, it can be concluded that it is possible, albeit with different efficiency, to synthesize geraniol esters using whole-cell biocatalysts and crude supernatant.

In the literature, there are examples of applications of whole-cell *Y. lipolytica* biocatalysts in ester synthesis reactions. The fragrance ester – 2-phenylethyl acetate was successfully synthesized by transesterification using biomass of the *Y. lipolytica* KKP 379 strain [34]. The freeze-dried biomass of these yeasts was also used for the synthesis of lipophilic esters of phenolic acids with antioxidant activity [12,13]. Moreover, whole yeast cells of the *Y. lipolytica* YL2 strain were used in the enantioselective hydrolysis of enol esters, as well as in the asymmetric resolution of racemic mixtures of secondary esters and prochiral γ-lactones [35].

### 3.5. Evaluation of Ester Biological Activity

Based on the structure of the substrates used for the synthesis of geranyl ester, it was suspected that one of them, namely 4-hydroxyphenylpropanoic acid, may exhibit antioxidant properties. Similarly, the non-esterified hydroxyl group of the phenolic ring in the ester molecule should predispose the obtained compound to radical scavenging activity. Therefore, it was decided to evaluate all reaction reagents for antioxidant activity. Two different methods were used to confirm whether the compounds used had antioxidant properties. In the first stage, the reduction of the DPPH**•** radical was investigated and the IC_50_ value was determined (Table 4). Based on the statistical analysis, it was shown that between 4-hydroxyphenylpropanoic acid, the obtained ester, and geraniol there is a significant difference in the reduction degree of DPPH**•** radical (32.22%, 28.71% and 2.43%). Hence, it has been concluded that the product and the substrate (phenolic acid), showed a similar antioxidant effect. Due to the fact that geraniol had a low ability to quench the radical, it was not taken into account when calculating the IC_50_ value. Table 4 shows the concentration values for the tested chemical compounds that reduce 50% DPPH**•**. It was found that there were no significant differences between the acid (81.20 mM) and the ester (90.25 mM), and these compounds showed similar antioxidant properties. To sum up, it was revealed that the novel compound – geranyl 4-hydroxyphenylpropanoate was characterized by similar antioxidant properties as its acid precursor.

Another method used—CUPRAC—confirmed the previously formulated conclusions, as the statistical analysis showed that there are significant differences in TEAC values between 4-hydroxyphenylpropanoic acid (0.746) and geraniol (0.045). The obtained ester exhibits moderate activity—0.513 in the method with water-ethanol environment. The potential of using derivatives of phenolic acids as natural antioxidants drew the attention of researchers due to their ubiquitous occurrence in nature. Phenolic acids have been extensively studied as potential antioxidants and are also added to food to prevent oxidation processes and for better food preservation—e.g., gallic acid derivatives (E310–E312). The relatively low solubility of phenolic compounds in non-polar media can be viewed as a disadvantage when considering their use as antioxidants to stabilize lipids or emulsions [36]. Enzymatic esterification allowed obtaining compound more soluble in organic solvents and environments, which was also confirmed by calculated partition coefficient, as well as the logarithm of solubility. Geranyl 4-hydroxyphenylpropanoate was the most lipophilic compound (cLogP = 5.25; cLogS = −3.41), followed by geraniol (cLogP = 3.49; cLogS = −1.89) and 4-hydroxyphenylpropanoic acid (cLogP = 1.25; cLogS = −1.57).

Another method used—CUPRAC—confirmed the previously formulated conclusions, as the statistical analysis showed that there are significant differences in TEAC values between 4-hydroxyphenylpropanoic acid (0.746) and geraniol (0.045). The obtained ester exhibits moderate activity—0.513 in the method with water-ethanol environment. The potential of using derivatives of phenolic acids as natural antioxidants drew the attention of researchers due to their ubiquitous occurrence in nature. Phenolic acids have been extensively studied as potential antioxidants and are also added to food to prevent oxidation processes and for better food preservation—e.g., gallic acid derivatives (E310–E312). The relatively low solubility of phenolic compounds in non-polar media can be viewed as a disadvantage when considering their use as antioxidants to stabilize lipids or emulsions [36]. Enzymatic esterification allowed obtaining compound more soluble in organic solvents and environments, which was also confirmed by calculated partition coefficient, as well as the logarithm of solubility. Geranyl 4-hydroxyphenylpropanoate was the most lipophilic compound (cLogP = 5.25; cLogS = −3.41), followed by geraniol (cLogP = 3.49; cLogS = −1.89) and 4-hydroxyphenylpropanoic acid (cLogP = 1.25; cLogS = −1.57).

Antibacterial activity of the phenolic acid, geraniol and obtained ester was compared and MIC and MBC values for four bacteria were determined (Table 5). 4-Hydroxyphenylpropanoic acid MIC values ranged 8–16 mM, and MBC values were at least 32. Similarly, for geraniol, these values were 8–16 mM, and 16–64 mM, respectively. In the case of geranyl 4-hydroxyphenylpropanoate, MIC values ranged 0.125–8. It was found that *B. subtilis* PCM 486 proved to be the most resistant bacteria. It was not possible to determine the sufficient concentration of tested compounds required to kill this strain. In contrast, *L. monocytogenes* was the most susceptible bacteria on the action of phenolic acid derivative.

In addition to MIC and MBC values, the ratios MBC/MIC were calculated, which allows for determination of whether the tested compounds exhibit bacteriostatic (ratio in the range of 1–2) or bactericidal activity (ratio of 4–16) [37]. In most cases, apart from the results for *B. subtilis*, it can be concluded that the compared compounds show bacteriostatic activity. It can be observed that esterification of phenolic acid with geraniol increased the antibacterial activity.

In the research of Zieniuk et al. [38], five different linear alcohols (C2–C10) were applied in the esterification of 4-hydroxyphenylpropanoic acid and the antibacterial activity increased with increasing the length of the alkyl chain. Similar observations were described by Merkl et al. [39]. The authors esterified protocatechuic, gentisic, ferulic, caffeic, vanillic and *p*-hydroxybenzoic acids with methanol, ethanol, propanol and butanol and tested obtained esters in antimicrobial assays. Similarly, increasing the lipophilicity of phenolic acids increased the antimicrobial activity and butyl esters were the most active compounds.

Esters of gallic acid behaved similarly. The series of alkyl gallic esters were evaluated by Zhang et al. [40] and Xu et al. [41]. In both studies, octyl gallate exhibited outstanding antimicrobial activity. It was acknowledged that the alkyl chain length played a significant role in inhibitory microorganisms’ growth. Zhang et al. [40] showed that edible chitosan-based films with octyl gallate significantly extended the shelf life of the Russian sturgeon. Moreover, the study of Xu et al. [41] revealed that the aforementioned ester may be an environmentally friendly wood preservative and sufficiently inhibited the growth of white-rot fungi.

## 4. Conclusions

In this study, the use of freeze-dried *Y. lipolytica* enzyme preparations allowed us to obtain geranyl 4-hydroxyphenylpropanoate-ester with antioxidant and antibacterial activity and although the obtained reaction yield was lower than that of CALB, it was possible to omit the multi-stage and costly process of isolating and purifying the enzymes. It is certainly one of the advantages of using whole-cell biocatalysts that they can be used directly for the reaction, without the need for purification and immobilization.

The current work has led to the conclusion that the freeze-drying is a useful method of enzyme preparation conservation and that low water activity along with using protective additives, like ammonium sulphate, could improve enzyme activity. The evidence from this study implies that *Y. lipolytica* could be a commercial source of lipases, but optimization of the idea is indispensable. Although our results are below expectations, the process can be improved and the further work should focus on loadings of lipase and introducing some simple partial purification steps, what can move on to growth in synthesis yield.

The obtained ester was more lipophilic than its precursor—4-hydroxyphenylpropanoic acid—due to the esterification with geraniol that cleared the way for its application in lipid-rich food matrices as an antioxidant agent or as an antimicrobial additive to limit the growth of unfavorable microflora of food products.

## Figures and Tables

**Table 1:** The water activity of supernatants after the freeze-drying process.

Characteristics of the Freeze-Dried Enzyme Preparation	Temperature of Secondary Drying (°C)	Water Activity a_w_
control sample—preparation without additives	10	0.160
control sample—preparation without additives	50	0.097
freeze-dried supernatant with the addition of 0.5% ammonium sulphate	10	0.141
freeze-dried supernatant with the addition of 0.5% ammonium sulphate	50	0.117
freeze-dried supernatant with the addition of 1% ammonium sulphate	10	0.156
freeze-dried supernatant with the addition of 1% ammonium sulphate	50	0.122
freeze-dried supernatant with the addition of 2% milk protein hydrolyzate	10	0.099
freeze-dried supernatant with the addition of 2% milk protein hydrolyzate	50	0.091
freeze-dried supernatant with the addition of 4% milk protein hydrolyzate	10	0.050
freeze-dried supernatant with the addition of 4% milk protein hydrolyzate	50	0.055
freeze-dried supernatant with the addition of 6% milk protein hydrolyzate	10	0.052
freeze-dried supernatant with the addition of 6% milk protein hydrolyzate	50	0.095

**Table 2:** The water activity of crude freeze-dried enzyme preparations incubated in controlled atmosphere with various water activities.

Water Activity in the Desiccator	Water Activity of the Freeze-Dried Enzyme Preparations
0.000	0.083
0.329	0.313
0.529	0.505
0.750	0.714

**Table 3:** The yield of the geranyl 4-hydroxyphenylpropanoate synthesis depending on the biocatalyst used.

Biocatalyst	Esterification Yield (%)
Lipase B from *C. antarctica*	73.4 ± 3.7 ^A^
Freeze-dried biomass of *Y. lipolytica* KKP 379	47.9 ± 1.9 ^B^
Freeze-dried supernatant of *Y. lipolytica* KKP 379	5.3 ± 0.3 ^C^

The values with a different letter (A–C) in a column are significantly different (α = 0.05).

**Table 4:** Comparison of antioxidant activity of tested compounds by means of DPPH**•** and CUPRAC methods.

Compound	DPPH• Method		CUPRAC Method
	Antioxidant Activity (%)	IC_50_ (mM)	TEAC
4-Hydroxyphenylpropanoic acid	32.22 ± 2.70 ^A^	81.20 ± 5.69 ^A^	0.746 ± 0.039 ^A^
Geranyl 4-hydroxyphenylpropanoate	28.71 ± 1.82 ^A^	90.25 ± 3.64 ^A^	0.513 ± 0.035 ^B^
Geraniol	2.43 ± 0.73 ^B^	n.a.	0.045 ± 0.003 ^C^

The values with a different letter (A–C) in a column are significantly different (α = 0.05).

**Table 5:** Comparison of antibacterial activity of synthesized ester and its precursor.

		4HPPA	Geraniol	G4HPP
*L. monocytogenes* PCM 2191	MIC (mM)	16	16	0.125
	MBC (mM)	32	16	0.5
	MBC/MIC	2	1	4
*B. subtilis* PCM 486	MIC (mM)	8	8	2
	MBC (mM)	>64	>64	>64
	MBC/MIC	>16	>16	>16
*K. pneumoniae* PCM 1	MIC (mM)	8	16	4
	MBC (mM)	32	32	32
	MBC/MIC	4	2	8
*E. coli* PCM 2057	MIC (mM)	16	16	8
	MBC (mM)	64	32	32
	MBC/MIC	4	2	4

Abbreviations: 4HPPA—4-Hydroxyphenylpropanoic acid; G4HPP—Geranyl 4-Hydroxyphenylpropanoate; MIC—Minimum Inhibitory Concentration; MBC—Minimum Bactericidal Concentration.

## Data Availability

The data presented in this study are available on request from the corresponding author (B.Z.).
